# Supplementary figures and images for: Structural analysis of viral ExoN domains reveals polyphyletic hijacking events
Source: PLoS One. 2021 Mar 17;16(3):e0246981. doi: 10.1371/journal.pone.0246981 (PMC7968707; doi:10.1371/journal.pone.0246981)

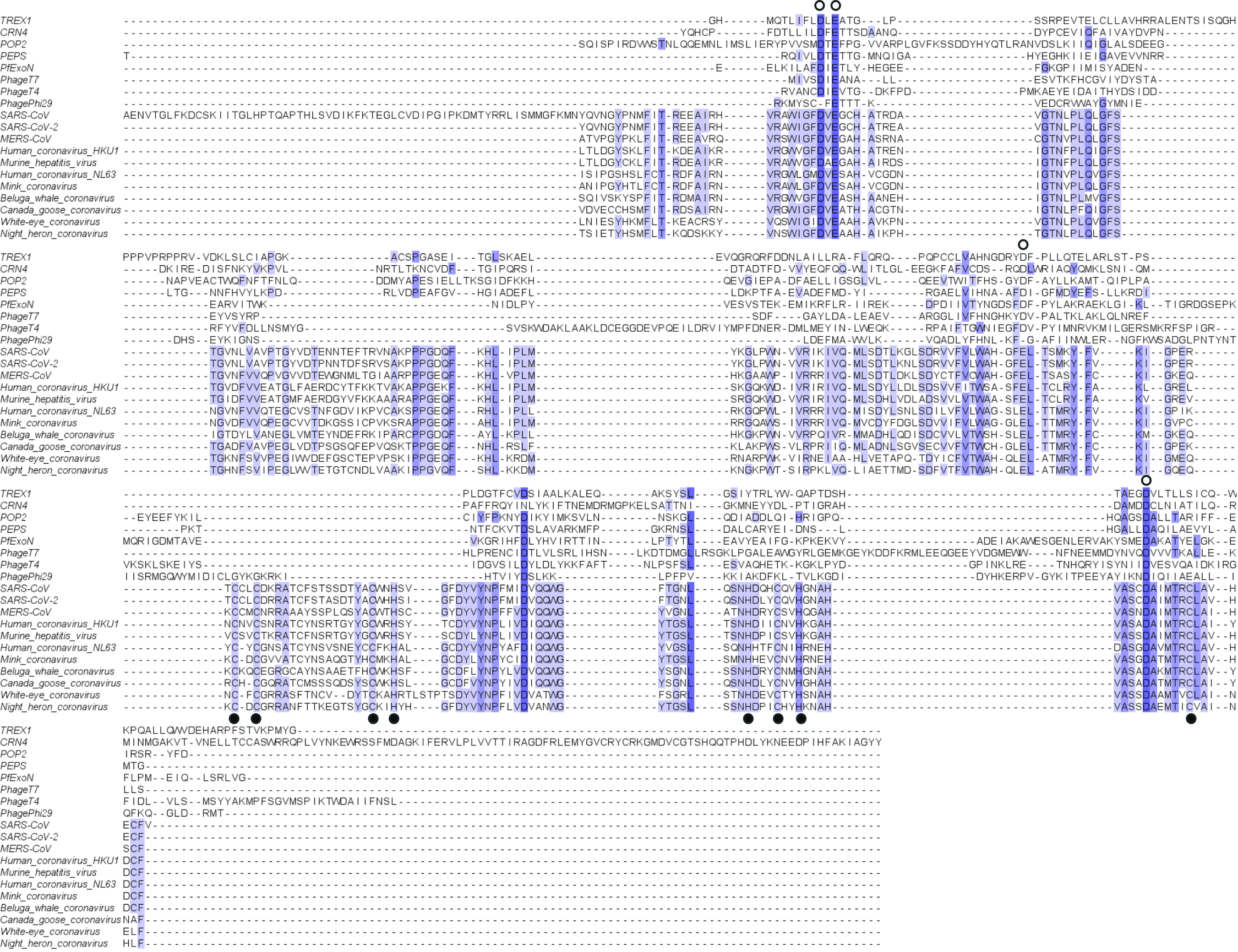

Supplement: S1 Fig — In SARS-CoV ExoN structure, Exo I (DE), Exo II (D/E), and Exo III (D) conserved sequence motifs are highlighted in green. Zinc-binding motif 1 (ZF1, CCCH/C) and zinc-binding motif 2 (ZF2, HCHC) are highlighted in red. Zn2+ is depicted as dark grey spheres and Mg2+ as a yellow sphere. In the logo, Exo I, Exo II, and Exo III are signaled with green arrows, while ZF1 and ZF2 are signaled with red arrows. Logo was made with WebLogo 3 (http://weblogo.threeplusone.com/). (TIF) [file pone.0246981.s001.tif]

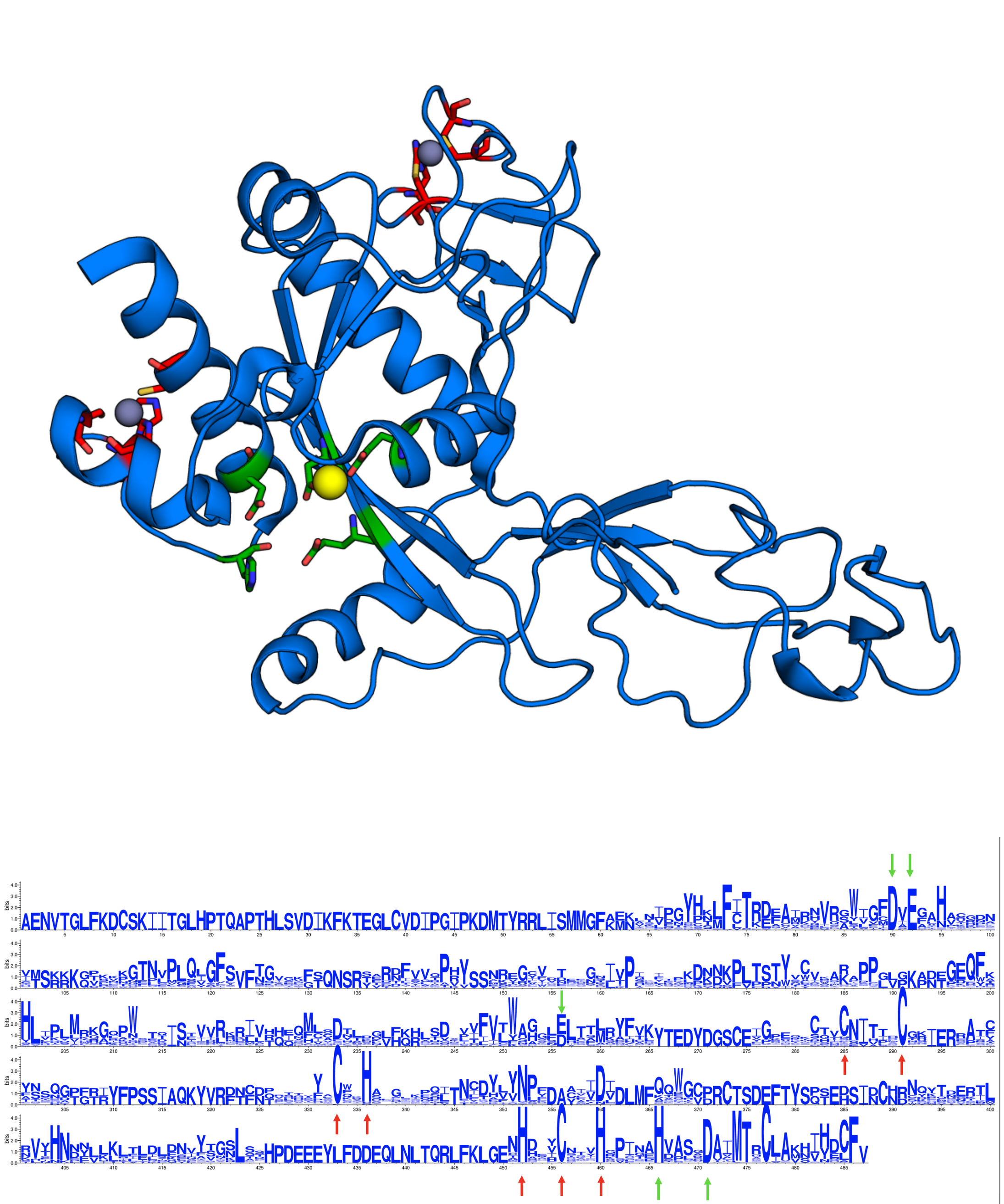

Supplement: S2 Fig — The Exo I (DE), Exo II (D/E), and Exo III (D) conserved sequence motifs are signaled with unfilled circles. Zinc-binding motif 1 (ZF1, CCCH/C) and zinc-binding motif 2 (ZF2, HCHC) are signaled with filled circles. Due to experimental procedures, the DEDD catalytic residues of the Bacillus virus phi29 ExoN domain (PhagePhi29) were mutated, showing a AADD sequence motif (the mutated residues are depicted with gaps in this alignment). (TIF) [file pone.0246981.s002.tif]
