# Supplementary material for: Structural analysis of viral ExoN domains reveals polyphyletic hijacking events
Source: PLoS One. 2021 Mar 17;16(3):e0246981. doi: 10.1371/journal.pone.0246981 (PMC7968707; doi:10.1371/journal.pone.0246981)
Supplement: S1 Table — (DOCX) [file pone.0246981.s003.docx]

**S1 Table. PDBs of the DnaQ-like exonucleases used in this work.**

| **PDB code** | **Organism** | **Abbreviation** |
| --- | --- | --- |
| 5C8U | *SARS-CoV* | SarsEXON |
| 1Y97 | *Homo sapiens* | HsapTREX2 |
| 3B6O | *Mus musculus* | MmusTREX1 |
| 3CG7 | *Caenorhabditis elegans* | CelgCRN4 |
| 2XRI | *Homo sapiens* | HsapERI3 |
| 2IDO | *Escherichia coli* | EcolPEPS |
| 5Z9X | *Arabidopsis thaliana* | AthaSDN1 |
| 2P51 | *Saccharomyces pombe* | SpomPOP2 |
| 1W0H | *Homo sapiens* | HsapERI1 |
| 4KAZ | *Escherichia coli* | EcolRIBT |
| 1WLJ | *Homo sapiens* | HsapISG2 |
| 4RG8 | *Methylocaldum szegediense* | MszeEXO1 |
| 4FZX | *Escherichia coli* | EcolEXOX |
| 1FXX | *Escherichia coli* | EcolEXO1 |
| 6A4A | *C. psychrerythraea* | CpsyOLRN |
| 4CZW | *Neurospora crassa* | NcraPAN2 |
| 4NLB | *Trypanosoma brucei* | TbruRRP6 |
| 6RCL | *Homo sapiens* | HsapREX2 |
| 2FBX | *Homo sapiens* | HsapWEXO |
| 1D9D | *Escherichia coli* | EcolKLFR |
| 1YT3 | *Escherichia coli* | EcolRIBD |
| 3IAY | *Saccharomyces cerevisiae* | ScerPDEL |
| 4GV6 | *Lassa virus* | LvirNUCL |
| 4OKE | *Mycobacterium tuberculosis* | MtubRNAS |
| 1Q9X | *Escherichia phage RB69* | Ph69EXON |
| 5LRP | *Mopeia virus* | MvirNUCL |
| 2JGU | *Pyrococcus furiosus* | PfurPEXO |
| 4K7E | *Junin virus* | JvirNUCL |
| 4PTF | *Saccharomyces cerevisiae* | ScerDEPS |
| 1T7P | *Bacteriophage T7* | T7EXON |
| 1NOZ | *Bacteriophage T4* | T4EXON |
| 2EX3 | *Bacillus virus phi29* | Φ29EXON |
